# Fetal aortic coarctation: A combination of third-trimester echocardiographic parameters to improve the prediction of postnatal outcome

**DOI:** 10.3389/fped.2022.866994

**Published:** 2022-10-10

**Authors:** Giulia Tuo, Dario Paladini, Lucia Marasini, Silvia Buratti, Gabriele De Tonetti, Maria G. Calevo, Maurizio Marasini

**Affiliations:** ^1^Department of Surgery, Pediatric Cardiology and Cardiac Surgery, Istituto di Ricovero e Cura a Carattere Scientifico (IRCCS) Istituto Giannina Gaslini, Genova, Italy; ^2^Department of Critical Care and Perinatal Medicine Fetal Medicine and Surgery Unit, IRCCS Istituto Giannina Gaslini, Genova, Italy; ^3^Department of Neuroscience, Rehabilitation, Ophthalmology, Genetics, Maternal and Child Health (DINOGMI), IRCCS Istituto Giannina Gaslini, University of Genoa, Genova, Italy; ^4^Critical Care and Emergency Department, Neonatal and Pediatric Intensive Care Unit, IRCCS Istituto Giannina Gaslini, Genova, Italy; ^5^Department of Critical Care and Perinatal Medicine, Obstetric Anesthesia, IRCCS Istituto Giannina Gaslini, Genova, Italy; ^6^Epidemiology and Biostatistics Unit, Scientific Direction, IRCCS Istituto Giannina Gaslini, Genova, Italy

**Keywords:** coarctation of the aorta, prenatal diagnosis, cardiovascular disproportion, fetal echocardiography, risk stratification

## Abstract

**Objectives:**

This study aims to determine a combination of third-trimester echocardiographic parameters for improving the prenatal prediction of coarctation of the aorta (CoA) after birth.

**Methods:**

We included all cases of suspected CoA during fetal echocardiography performed in the second and/or third trimester of pregnancy at Gaslini Children's Hospital between January 2010 and December 2020. The last prenatal ultrasound evaluation was reviewed considering most of the echocardiographic criteria were already published for prenatal CoA diagnosis. Associated minor cardiac anomalies, such as a ventricular septal defect, persistent left superior vena cava (PLSCV), and redundant foramen ovale (FO) membrane, as well as postnatal outcomes, were reported. Initial perinatal management was defined based on the risk stratification of CoA during prenatal echocardiography. Neonates were divided into two groups depending on the presence or absence of CoA after birth.

**Results:**

A total of 91 fetuses with CoA suspicion were selected, of which 27 (30%) were confirmed with CoA after birth and underwent surgical repair. All cardiac parameters except redundant FO membrane and PLSCV showed a significant correlation with CoA. Statistical analysis confirmed that cardiovascular disproportion with right predominance carries an increased risk for occurrence of CoA, especially if already evident during the ultrasound evaluation in the second trimester. Aortic valve (AV) z-score and distal transverse aortic arch (TAA) z-score resulted as the best predictors of CoA after birth. The best cutoff point for CoA discrimination with ROC analysis was an AV z-score of −1.25 and a distal TAA z-score of −0.37. A total of 46% of those without CoA were diagnosed with a cardiac defect, which was not diagnosed in utero, pulmonary hypertension, or a genetic syndrome.

**Conclusion:**

The current criteria for diagnosing CoA *in utero* allow accurate diagnosis of most severe cases but the rate of false positives remains relatively high for milder cases. A combination of anatomic and functional echocardiographic parameters might be used in stratifying the risk of CoA. We proposed the AV and the TAA diameter z-scores as the best predictors of CoA after birth. In addition, neonates without CoA deserve proper monitoring at birth because prenatal evidence of a significant cardiovascular discrepancy between the right and left cardiac structures has an inherent risk for additional morbidity postnatally.

## Introduction

The coarctation of the aorta (CoA) is the narrowing of the thoracic aorta in the region of the insertion of the arterial duct, with or without additional abnormalities of the aortic arch (1). CoA occurs in 7–8% of newborn children with congenitally malformed hearts and is a cause of remarkable morbidity and mortality if not diagnosed early (1, 2). Upon birth and after ductal closure, a critical CoA will cause an increase in left ventricular afterload with severe cardiac failure and poor perfusion of the lower body (3).

It has already been demonstrated that the diagnosis of prenatal CoA improves perioperative condition and survival of affected neonates by allowing planned delivery in a tertiary care center and early institution of prostaglandin treatment to prevent the closure of the duct (4, 5). However, *in utero* diagnosis of CoA still remains challenging and is affected by a high rate of false positives, especially during the third trimester, because it relies on indirect and non-specific signs, such as cardiac asymmetry with right dominance that may also be seen in normal fetuses in late pregnancy (6).

Several echocardiographic measurements have already been proposed to improve the detection rate of prenatal diagnosis of CoA, including two-dimensional measurements of the ventricular inflow and outflow tracts and the derived z-scores, the comparison of left and right side structure diameters, Doppler signs, and other features like the presence of a persistent left superior vena cava (LSVC), the visualization of a juxtaductal shelf, and the carotid subclavian index (7–18).

This study aimed to review the institutional experience in the prenatal diagnosis and the perinatal and postnatal management of fetuses with the suspicion of CoA. We also sought to confirm the usefulness of several previously published echocardiographic predictors of postnatal CoA, especially in those fetuses referred to a tertiary care center from an extra-regional care facility during late pregnancy.

## Methods

All cases of suspected CoA detected at fetal echocardiography in the second and/or third trimester of pregnancy at Gaslini Children's Hospital between January 2010 and December 2020 were retrieved from our Cardiology Department database. The suspicion of CoA was based on prenatal features consistent with this condition, including the evidence of cardiovascular disproportion, which is defined as the discrepancy in the size of either cardiac chambers or great vessels detected during the ultrasound evaluation (7).

All transabdominal fetal echocardiograms including pulsed, continuous, and color Doppler examination (iE33, Philips, Amsterdam, The Netherlands) were reviewed offline. The retrospective analysis was carried out by two authors (G.T. and M.M.) on stored videos imported into the Xcelera Philips R3.3L1 software platform for offline analysis. Gestational age (GA) was determined by the last menstruation. Fetal heart examination routinely included the transverse view of the upper abdomen, the four-chamber view, the three-vessel view, the left and right ventricular outflow tract view, and the aortic arch view.

**Figure 1:**
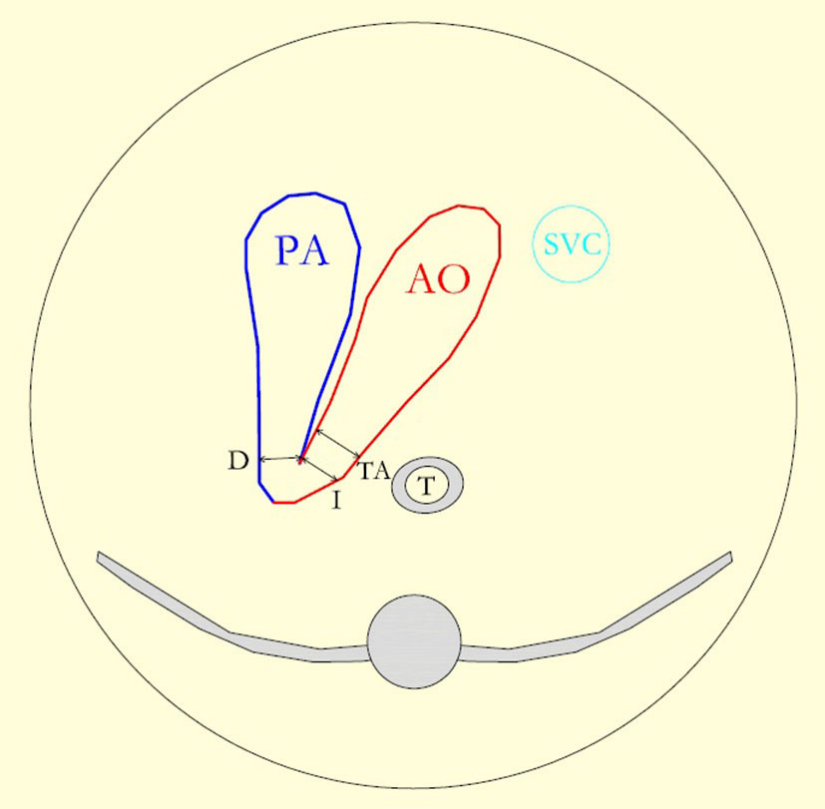
The diagram reproduces the three vessel and the tracheal view and shows the pulmonary artery (PA) leading into the arterial duct (D), the aortic arch (AO), and the superior vena cava (SVC). The lines show the measurement of, respectively, the isthmus (I), the duct, and the distal transverse aortic arch (TA) diameter.

The last prenatal echocardiography, which was performed during the third trimester of pregnancy, was reviewed taking into consideration most of the echocardiographic criteria already published for the prenatal CoA diagnosis (7–18). The following retrospective measurements were carried out: mitral valve (MV) z-score; mitral/tricuspid valve (MV/TV) ratio; left/right ventricular (LV/RV) diameter ratio; aortic valve (AV) z-score; aortic/pulmonary valve (AV/PV) ratio; main pulmonary artery/ascending aorta ratio (MPA/AA); distal transverse aortic arch (TAA) and aortic isthmus diameter (AI) z-score; aortic isthmus/arterial duct (AI/AD) ratio. Cardiac dimensions were measured at their maximum size and measurements were taken from the inner edge to the inner edge. The diameter of the atrioventricular valves was measured in an apical four-chamber view. The internal diameter of the aortic isthmus was measured immediately proximal to the insertion of the arterial duct in the three vessels and in tracheal view. The ductal diameter and the distal transverse aortic arch diameter were also acquired in the three vessels and in tracheal view (Figure 1) (15). The subjective appearance of both the aortic arch (either diffuse hypoplasia of the whole aortic arch or not) and the left ventricle (“borderline” left ventricle or adequate size) was reported. The appearance of the foramen ovale was studied, and the foramen ovale membrane was defined as redundant when it herniated into the left atrium for more than 50% of the left atrial diameter (19). GA was used to calculate the z-score (20). Color Doppler assessment of flow, respectively, at the aortic arch (antegrade flow alone or antegrade and reversed ductal-dependent flow) and at the foramen ovale (right to left or bidirectional) were recorded. Fetuses with associated minor cardiac anomalies, such as ventricular septal defect (VSD), bicuspid aortic valve suspicion, persistent left superior caval vein (PLSCV), and redundant foramen ovale (FO) membrane, were included in the study. Fetuses with hypoplastic left heart syndrome class IV, isolated aortic valve stenosis, aortic arch interruption type B or C, or other complex heart defects were not included in the study. We also excluded fetuses with unknown postnatal echocardiogram and/or outcomes.

Medical records were also reviewed for the following: indication for referral to a specialized tertiary care center, mean gestational age at the last fetal echocardiographic evaluation, associated extra-cardiac anomalies (ECA) or syndromes, karyotyping result, if performed, and postnatal diagnosis and outcomes.

Echocardiographic parameters acquired during the last fetal echocardiogram were compared between patients with confirmed surgical CoA and those without CoA detected at the postnatal ultrasound examination.

The risk of developing CoA after birth was stratified into low, moderate, or high based on obstetrical history (i.e., family history of CoA, early suspicion of cardiac asymmetry during the ultrasound scan in the second trimester) and major echocardiographic parameters detected at the last fetal echocardiographic examination. Low CoA risk fetuses received the first diagnosis of CoA suspicion during the third trimester of pregnancy (> 28 weeks of GA, late diagnosis), had an aortic isthmus narrowing (-3 < isthmus z-score ≤ -2; 0.5 < AI/AD ratio < 0.7) but a good size transverse aortic arch at the subjective analysis, and a mild discrepancy between the right and left side structures (LV/RV ratio ≥0.6). Moderate CoA risk fetuses received an early diagnosis (< 28 weeks of GA), had an aortic isthmus narrowing (isthmus z-score ≤ -3; AI/AD ratio ≤ 0.5), and a more evident discrepancy between left and right side dimensions both at the level of the ventricles (mitral z-score < -3, LV/RV ratio < 0.6) and of the great arteries (MPA/AA ratio 2) compared to the low CoA risk fetuses. High-risk fetuses presented moderate-risk anatomic features with marked hypoplasia of the aortic arch (distal TAA z-score < -1), a borderline LV with MV hypoplasia (z-score < -5), and an aortic arch flow reversal and bidirectional flow at the level of the foramen ovale.

Delivery of all neonates with prenatal CoA suspicion, regardless of the CoA risk degree, was planned at our institute, which is a major pediatric tertiary care center where high-risk pregnancies are centralized and also includes cardiac and cardiothoracic surgery services. Initial perinatal management was defined based on the prenatal risk stratification performed by the fetal cardiologist.

All neonates with a low risk of CoA at birth received a daily physical examination by pediatricians with palpation of femoral pulses, measurement of oxygen saturation, and systolic right brachial–right ankle pressure difference in a nursery setting. Echocardiography was performed by a pediatric cardiologist always between 48 and 72 h after birth. Those with a moderate or high risk of CoA were transferred at birth to the cardiac unit and received clinical monitoring and a echocardiogram respectively within first 12 hours or 4 hours after birth according to the estimated prenatal risk. Then, serial echocardiograms were performed until the diagnosis of CoA resulted in positive or negative confirmation. We did not place any umbelical venous arterial catheter and did not commence prostaglandin E1 (PGE1) until we had the confirmation of neonatal CoA.

Coarctation of the aorta was confirmed by postnatal echocardiography or CT. The diagnosis of CoA usually relies on the presence of a localized narrowing of the thoracic aorta just beyond the origin of the left subclavian artery, owing to a posterior shelf protruding from the posterior aspect of the aorta, together with the high-velocity flow (> 2 m/s) across the stenosis. Associated findings, such as isthmus and aortic arch hypoplasia, were diagnosed if the z-score was < -2, according to normal reference ranges (21).

Assessment of postnatal outcomes included the following: postnatal evidence of associated cardiac and extracardiac anomalies, chromosome or genetic disorders, presence of persistent neonatal pulmonary hypertension (PNPH), drug administration, need for one or more interventions, and/or age and cause of death, at least within the first 6 months of life. Neonates who were not diagnosed with CoA at birth underwent a cardiologic follow-up, including echocardiography at 1 and 6 months of life to rule out a late CoA.

Persistent neonatal pulmonary hypertension was defined as a failure of the normal circulatory transition that occurs after birth with the progressive reduction of pulmonary vascular resistance. The severity of PNPH was based on its duration and the need for respiratory support and medications. PNPH was defined as self-limiting (lasting less than 6 weeks) or persistent (lasting more than 6 weeks) (22, 23).

This study was approved by our institutional ethics committee, and informed consent to view files and echocardiographic reports was obtained from the parents.

### Statistical analysis

Descriptive statistics were generated for the whole cohort and data were expressed as mean and SD for continuous variables. Median value and range as well as absolute or relative frequencies for categorical variables were calculated and reported. Demographic and clinical characteristics were compared using the χ^2^ or Fisher's exact test and the Mann-Whitney *U* test for categorical and continuous variables, respectively. A univariate analysis was carried out to determine characteristics that were significantly more frequent among the patients with CoA. Logistic regression analyses were used for each variable, and the results were reported as odds ratio (OR) with their 95% confidence intervals (CIs). A multivariate analysis was then performed, and only variables that proved to be statistically or borderline significant in the univariate analysis (*P* < 0.08) were included in the model. The model showing the best fit was based on backward stepwise selection procedures, and each variable was removed if it did not contribute significantly. In the final model, a *p*-value of < 0.05 was considered statistically significant, and all *p*-values were based on two-tailed tests. Receiver operating characteristic (ROC) curves and area under the curve (AUC) were calculated for echocardiographic parameters significantly associated with the postnatal development of CoA. Cutoff points with corresponding sensitivity and specificity are provided. Statistical analysis was performed using Statistical Package for the Social Sciences (SPSS) for Windows (SPSS Inc., Chicago, IL).

## Results

Between January 2010 and December 2020, 102 consecutive fetuses with a suspicion of CoA in the second or third trimester of pregnancy were identified. Six pregnancies were terminated: three for the development of Shone's syndrome, two for CoA suspicion in Turner's syndrome, and one for CoA associated with hydrops fetalis. Five fetuses were lost at prenatal cardiac follow-up. The study population included 91 newborn fetuses (Table 1). Of which, 27 (30%) were confirmed with CoA after birth and underwent surgical procedures within the first month of life. CoA risk was prenatally estimated to be high in 22 out of 27 of them and moderate in 5 out of 27. Among the other 64/91 fetuses that did not develop CoA after birth, 24/64 were prenatally considered to have a moderate CoA risk and 40/64 a low CoA risk.

**Table 1:** Prenatal details of the study population (fetuses with suspected coarctation of the aorta, CoA).

**Variable**	**Value**
Fetal study population	91
Type of pregnancy	
Single	89
Twin	2
Referral reason	
Suspected cardiac asymmetry	71
Suspected cardiac anomaly (VSD, PLSCV, Redundant FO Membrane)	8
Family medical history	7
Extracardiac anomaly	4
Chromosomal anomaly	1
Associated minor cardiac anomalies	49
Persistent left superior vena cava	10
CoA Risk assignment at last fetal echocardiography	
Low	39
Moderate	29
High	23
Extracardiac anomalies	8
Labiopalatoschisis	1
Congenital cystic adenomatoid malformation	1
Micropenis	1
Single umbilical artery	1
Renal dysplasia and anhydramnios	1
Polimalformative complex	1
Spinal Dysraphism	1
Esophageal atresia and Polyhydramnios	1
Karyotype analysis	23
Chromosomal abnormalities	3
Turner syndrome or monosomy 45 X	1
Partial deletion of short arm of chromosome 5	1
Partial Deletion of short arm of chromosome 4	1

**Figure 2:**
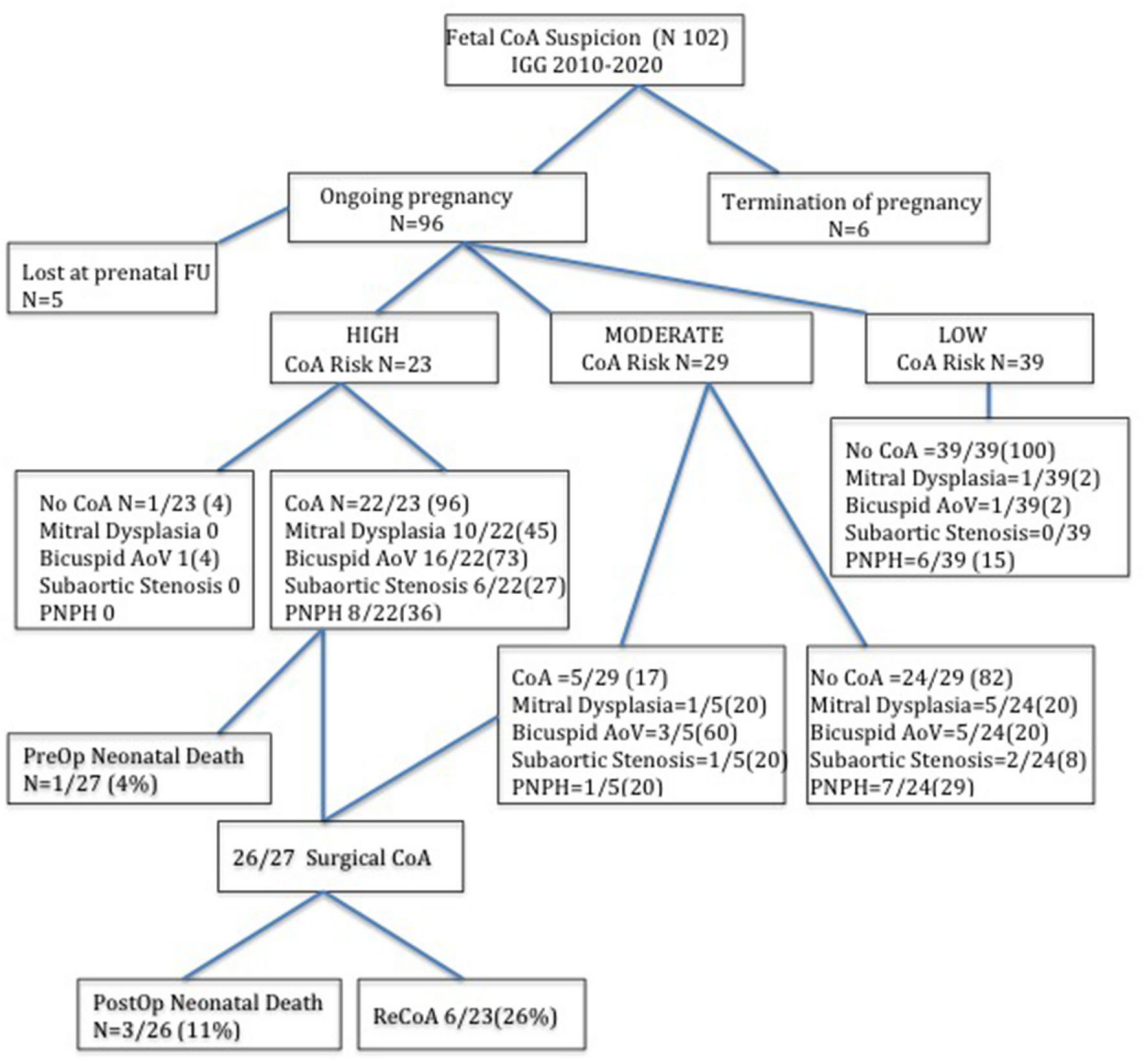
Flow chart of fetuses with suspected coarctation of aorta according to the prenatal risk stratification at last fetal echocardiography and the postnatal findings. The number between brackets indicates the percentage.

All neonates with a prenatal diagnosis of high-risk CoA suspicion required aortic arch repair except in one case that deserved further clinical and echocardiographic evaluation because of a bicuspid aortic valve and a VSD at the ultrasound examination of the neonate. Accordingly, surgical CoA was much more common in high (22/23, 96%) than moderate-risk CoA fetuses (5/29, 20%) and was never demonstrated in mild-risk CoA fetuses (0/39). There was no significant difference in the gestational age between different CoA risk categories (Figure 2).

Among the high and moderate CoA risk fetuses that developed surgical CoA (27/91), we also found mitral dysplasia in 11 cases (11/27, 40%) and a bicuspid valve in 19 cases (19/27, 70%) cases. Nine out of 27 (33%) neonates showed PNPH that was self-limiting but required temporary respiratory support. Following echocardiographic evaluation, we detected mild subaortic stenosis in 7 out of 27 neonates (26%). Three neonates died after the surgical procedure (3/27, 11%), of whom two had a complex form of CoA associated with Shone's syndrome. One more neonate with CoA was diagnosed with the CHARGE Syndrome postnatally and died before surgical correction. During the subsequent clinical and echocardiographic follow-up, 6 out of 23 (26%) neonates showed re-CoA and underwent percutaneous dilatation by balloon angioplasty.

Among the moderate and low-risk CoA patients that did not develop CoA (63/91), one was postnatally diagnosed with superior sinus venosus atrial septal defect and partial anomalous pulmonary venous drainage of the right upper pulmonary veins into the superior vena cava and underwent surgical correction. One patient was diagnosed with Cardiospondylocarpofacial syndrome after birth. Moreover, out of 63, 6 (9.5%) of them were found to have mitral dysplasia, 6 (9.5%) had a bicuspid aortic valve, and 5 (8%) had a diffuse hypoplastic aortic arch. One patient with a bicuspid aortic valve subsequently developed a moderate degree of aortic valve stenosis and ascending aorta dilatation that did not require any interventional procedure until now. Two out of 63 patients developed subaortic stenosis during the follow-up. In total, 13 out of 63 (21%) neonates without CoA needed temporary respiratory support for self-limiting neonatal PNPH.

Therefore, only 34 out of 91 (37%) patients with prenatal CoA suspicion did not show any cardiac or extracardiac anomaly after birth, and they belonged to the prenatal low and moderate-risk CoA groups.

**Figure 3:**
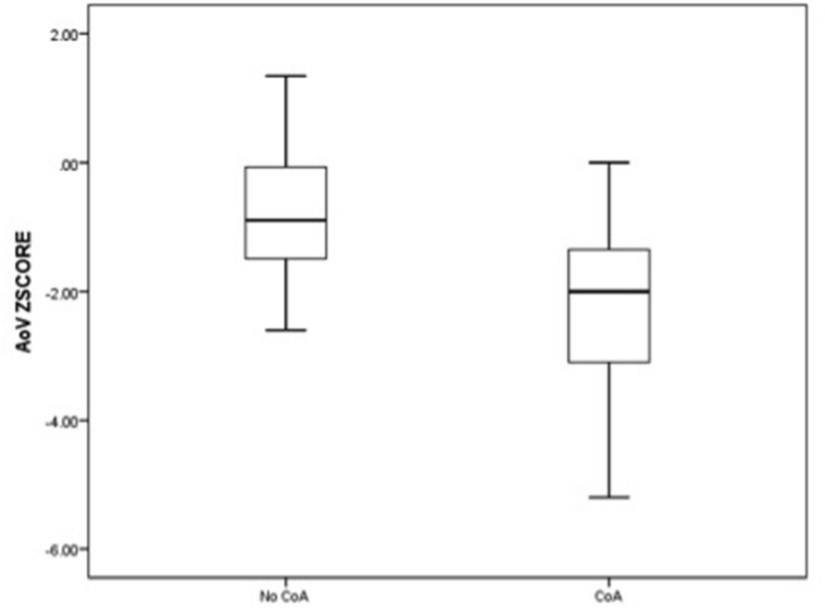
Box and whisker plots of the z-score, respectively, for aortic valve annulus and the distal transverse aortic arch at the third-trimester echocardiography.

**Figure 4:**
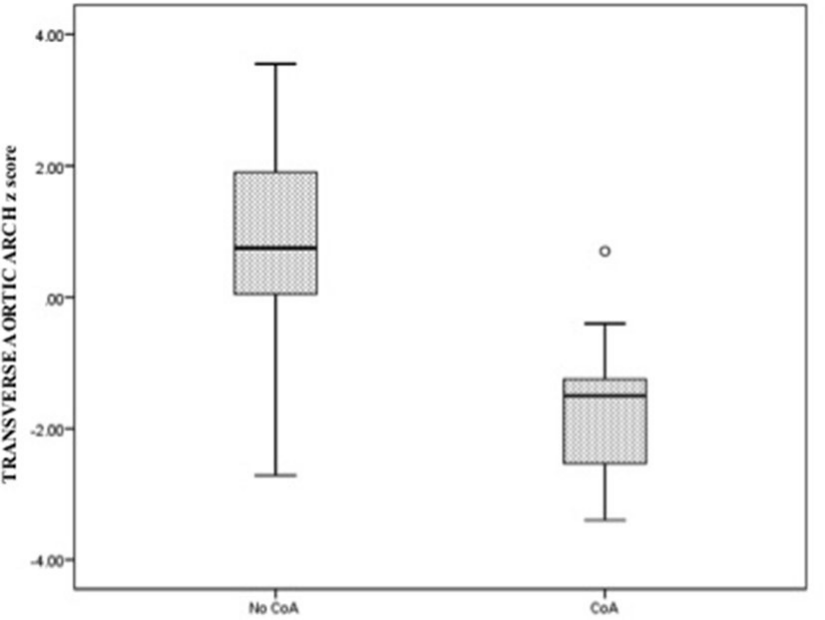
Box and whisker plots of the z-score, respectively, for aortic valve annulus and the distal transverse aortic arch at the third-trimester echocardiography.

The comparative analysis between newborns with confirmed or without confirmed surgical CoA is detailed in Table 2. There was no significant difference in gestational age between the two groups. In the univariate analysis, all parameters were statistically significant except for the LSCV. The mean MV diameter z-score and the LV/RV ratio were significantly lower in fetuses with CoA than in those without CoA. Furthermore, the mean aortic valve diameter z-score was significantly lower in fetuses with CoA. In contrast, MPA/AA ratio was significantly higher in fetuses with CoA. Mean aortic isthmus diameter z-score and mean distal transverse aortic arch z-score were significantly lower in fetuses with CoA than in those without CoA, and the subjective impression of a diffuse hypoplastic aortic arch was associated with the occurrence of CoA (Figures 3, 4). Whereas, the presence of a redundant foramen ovale resulted in a protective feature against the development of surgical CoA after birth.

**Table 2:** Prenatal ultrasound findings of the study population (fetuses with suspected CoA), according to postnatal outcomes (neonates normal and with CoA).

**Variable all patients** ***N =* 91**	**No CoA** ***N =* 64**	**CoA** ***N =* 27**	***p*-value**
GA at last fetal ECHO	35.29 (2.18)	35.48 (2.24)	0.72
Early CoA diagnosis ( ≤ 28 weeks)	16 (25%)	23 (85.2%)	0.0001
Borderline LV	0	10 (37%)	0.0001
Inflow tracts			
z-score MV	−2.67 (1, 02)	−3.93 (1, 30)	0.0001
MV/TV ratio	0.65 (0.09)	0.51 (0.08)	0.0001
LV/RV ratio	0.64 (0.08)	0.53 (0.11)	0.0001
Outflow tract			
z-score AV	−0,74(0.94)	−2.22 (1.27)	0.0001
AV/PV ratio	0,75 (0.30)	0,56 (0.09)	0.0001
MPA/AA ratio	1,52 (0.33)	1,88 (0.26)	0.0001
Aortic Arch (on three vessel view)			
AI z-score	−2.26 (1.14)	−3.59 (1.12)	0.0001
Hypoplastic TAA	9 (14%)	26 (96%)	0.0001
TAA z-score	0.71 (1.35)	−1.76 (0.94)	0.0001
AI/AD ratio	0.51 (0.13)	0.43 (0.1)	0.009
Functional features			
Reversed or mixed flow at the aortic arch	17 (26.6%)	13 (48.1%)	0.05
Bidirectional flow at the foramen ovale	8 (12.5%)	17 (63%)	0.0001
Associated cardiac anomalies			
VSD	9 (14.1%)	9 (33.3%)	0.05
Redundant FO membrane	26 (40.6%)	5 (18.8%)	0.05
LSCV	7 (17.9%)	3 (15%)	1
Bicuspid aortic valve suspicion	5 (8%)	10 (37%)	0.0001
Quantitative results are expressed as mean (standard deviation)	

Univariate analysis.

Considering the significant variables in a multiple logistic regression analysis revealed that only AV z-score, LV/RV diameter ratio, and TAA z-score were statistically significant. Therefore, a higher measure of AV z-score, LV/RV diameter ratio, and TAA z-score resulted in protection against surgical CoA diagnosis after birth (Table 3).

**Table 3:** Multivariate logistic regression analysis of potential risk factors for CoA.

	**No CoA**	**CoA**	***OR (95%CI)***	***p-value***
	*N =* 64	*N =* 27		
AV z-score	−0,74 (0.94)	−2.22 (1.27)	0.27 (0.09–0.80)	**0.02**
LV/RV diameter	0.64 (0.08)	0.53 (0.11)	0 (0–0.06)	**0.01**
TAA z-score	0.71 (1.35)	−1.76 (0.94)	0.22 (0.10–0.48)	**0.0001**

*OR*, odds ratio; *CI*, confidence interval.

**Figure 5:**
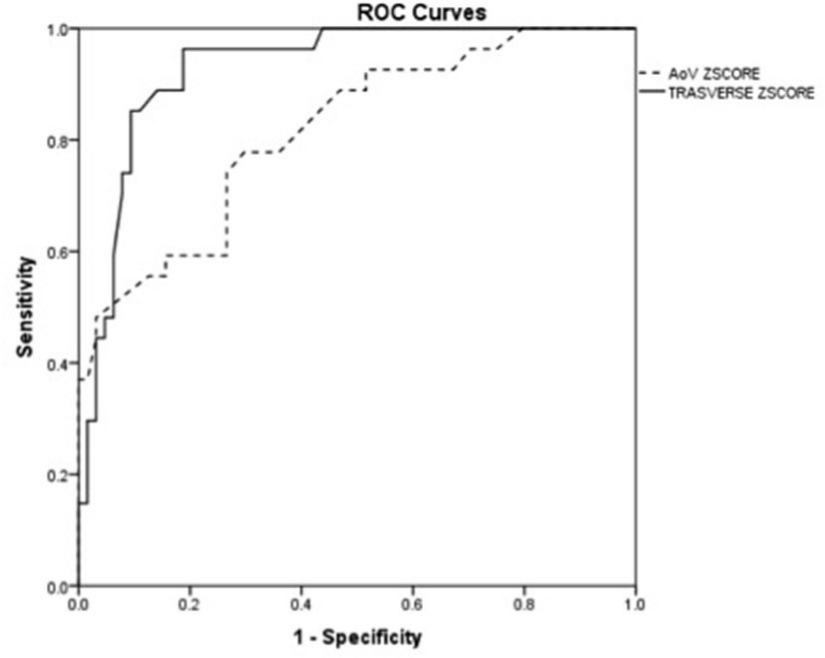
The receiver operating characteristic curve of the z-score aortic valve annulus and the z-score distal transverse aortic arch z-score at the third-trimester echocardiography.

We selected the two echocardiographic parameters that showed the strongest association with postnatal CoA, respectively, AV and distal TAA z-scores, and we presented data related to specificity and sensitivity, as well as cutoff points related to the ROC curves. The best cutoff point for CoA discrimination with ROC analysis was an AV z-score of −1.25 and a distal TAA z-score of −0.37. The value of the area under the curve relative to the AV z-score was 0.82 while for the variable distal TAA z-score was 0.93. The parameter with the greatest sensitivity and specificity was the distal TAA z-score ≤ -0.37 with 96.3 and 81.2%, respectively (Table 4, Figure 5).

**Table 4:** Cutoff points, sensitivity, specificity, and 95% confidence interval (CI) for CoA.

	**Cut-off value**	**Sensitivity (%) (95% CI)**	**Specificity (%) (95% CI)**
AV z-score	≤ -1.25	77.8 (62.1; 93.5)	70.3 (59.1; 81.5)
TAA z-score	≤ -0.37	96.3 (89.2; 100)	81.2 (71.7; 90.8)
			
AV z-score + TAA z-score	≤ -1.25 or ≤ -0.37	100 (100; 100)	56.2 (44.1; 68.4)

The left carotid to left subclavian artery (LCSA) distance was not retrievable for all fetuses at the retrospective analysis but it was measured at postnatal ultrasound examination in all patients, and it resulted significantly (*p* < 0.0001) higher in those who developed CoA (median 7; minimum 0.9 mm; maximum 11 mm) than in those without CoA (median 4 mm ±1.16; minimum 1.4, maximum 7.5 mm).

## Discussion

Coarctation of the aorta is one of the most common congenital heart defects and is life-threatening if undiagnosed (24). As already demonstrated, prenatal diagnosis of CoA may improve survival and the preoperative condition of neonates presenting for surgery (4). However, CoA is still challenging to diagnose *in utero* despite advances in prenatal screening for congenital heart defects and the application of detailed techniques that combine measurements of cardiac structures and Doppler analysis at the time of fetal echocardiographic evaluation (7–18). Moreover, pulse oximetry screening is misdiagnosed in up to 80% of cases of CoA before discharging home (25–27), and the delayed diagnosis increases morbidity and mortality of affected neonates.

Due to the pre- and post-natal screening system limits, many institutions adopted a cautious postnatal management plan once antenatal suspicion is raised, although contributing to the increase in false positive cases. This is warranted considering the severity of the disease if left undiagnosed, but the unnecessary admission of the neonate with the suspicion of CoA to the cardiac or neonatal intensive care unit may contribute to family distress due to the increased medicalization of an otherwise normal infant (27, 28).

Reviewing our experience relative to the last 11 years of diagnosis and management of fetuses with CoA suspicion, we confirmed that the specificity of prenatal CoA diagnosis is still poor with a high false positive rate. The accuracy of our impression in predicting the need for postnatal arch repair was high for cases that were highly likely to develop CoA (96% of true positive cases) but poor in fetuses with moderate (20% of true positive cases) and low risk of CoA (0/39 cases).

Our results were in concordance with Sharland et al.'s study in 1994 when they reported their experience at Guy's Hospital in London (6). The most severe forms of CoA were associated with relative hypoplasia of the left heart structures compared with the right, detected already in early pregnancy. On the contrary, the milder forms of CoA were consistent with a normal early fetal echocardiogram and with the evidence of a ventricular size discrepancy only during the ultrasound screening in the third trimester. Whereas in late pregnancy, the right heart structure may appear larger than the left in the normal fetus, and the examination of all the available echocardiographic parameters may not always be sufficient to distinguish between the real and the false positive cases. This has to be considered an inherent limitation of prenatal diagnosis.

Our study thus confirms the need for a risk-stratified approach to the neonate with prenatally diagnosed CoA suspicion, aiming to maximize the sensibility of true positives cases while minimizing morbidity of false positives.

Many echocardiographic parameters have been suggested until now to potentially improve the detection rate of CoA and the risk stratification process of CoA. A recent review published in circulation in 2017 demonstrated that fetuses with CoA have significant differences in several parameters, particularly in the left inflow and outflow tracts. Evidence of the hypoplastic aortic arch led to the best prenatal predictor of postnatal CoA (7).

In our experience, the cardiovascular disproportion between the left and right cardiac structures and the predominance of the right carries an increased risk for the occurrence of CoA, especially if already evident during the ultrasound evaluation in the second trimester. The statistical analysis defined AV z-score and distal TAA z-score as the best predictors of CoA after birth. These parameters were shown to have the best balance between sensitivity and specificity for the diagnosis of CoA in fetuses with isolated cardiac asymmetry. When combined, these parameters contribute to increasing the sensibility of the prenatal ultrasound examination but with the concomitant expected reduction of the specificity.

We determined the best cutoff value for the AV valve z-score was −1.25 (based on gestational age). In our opinion, during the third trimester, measuring the diameter of the aortic valve appeared easy, reproducible, and more predictive than the measurement of aortic isthmus diameter, which is in contrast to the other findings (9, 10, 13, 15). Gomez-Montes et al. (12) already demonstrated that measuring aortic isthmus diameter is not helpful to identify true false positive cases in late pregnancy (>28 weeks of gestation) because it is normal to observe a gradual tapering of the normal aortic arch whose narrowest site is the distal isthmus (12). Professor A. Rudolph previously explained that, in late gestation, the volume flowing across the aortic isthmus is quite low and probably represents only about 8% of combined ventricular output (29).

In our experience, the diffuse hypoplastic aortic arch was strongly associated with the occurrence of CoA as reported by several authors before (11, 12). However, the definition of the arch as hypoplastic has always been based on subjective impressions and not on a specific cutoff value. We, therefore, focused on the measurement of the distal TAA diameter, and we derived the relative z-score, which resulted in the parameter with the greatest sensitivity and specificity. To the best of our knowledge, we reported for the first time the best cutoff value for the distal TAA diameter z-score (based on gestational age).

Previous studies used sagittal or coronal views of the aortic arch for the definition of arch morphology and dimension (8). We considered the three vessel and tracheal view as the ideal plane for the direct comparison of arches size of the aorta and duct and the aortic isthmus measurement. Moreover, the sagittal view of the aortic arch was often technically difficult to acquire during the last echocardiogram because of the unfavorable fetal position or the abdomen's bad acoustic window. For these reasons, we could not include in the statistical analysis the presence of a posterior shelf or the distal displacement of the left subclavian artery. However, the LCSA distance was measured at the postnatal echocardiogram in all neonates with prenatal CoA suspicion and resulted significantly higher in those who developed CoA, confirming to be a helpful tool to predict CoA both postnatally and prenatally, if the sagittal view of the arch is achievable.

As already demonstrated, also in our experience, both bidirectional flow across the foramen ovale and reverse flow in the aorta were significantly more frequent among patients with confirmed CoA after birth. However, none of the two characteristics were identified as strong prenatal CoA predictors in the multivariate logistic analysis. In fact, these functional parameters may also be detected in the presence of an isolated redundant foramen ovale membrane that may mimic fetal aortic coarctation, especially during the third trimester of pregnancy, increasing the rate of false positive CoA diagnosis (6, 30–32).

It has been hypothesized that PLSVC may increase the likelihood of CoA (33). In this study, the incidence of PLSVC was quite similar in fetuses that proved to have CoA and in normal fetuses. Our results confirm that this venous abnormality may induce cardiac asymmetry because coronary sinus dilatation decreases left ventricular filling, leading to a false-positive diagnosis of CoA (13).

The incidence of the bicuspid aortic valve was diagnosed frequently in patients with CoA after birth. However, the difficulty in reliable prenatal diagnosis of bicuspid aortic valve makes this well-recognized association less helpful prenatally. It is also noteworthy that neonates that did not develop CoA showed a higher incidence of the bicuspid aortic valve than in the general population.

We developed protocols for both low risk and moderate-to-high risk CoA at Gaslini Children's Hospital based on our growing experience in the management of neonates with prenatal CoA suspicion. The mothers of suspected neonates were indicated to deliver in our center and transfer the neonate to the cardiac unit or the nursery setting based on prenatal risk stratification. Our policy seeks to reduce the necessity of transferring a sick neonate from another hospital after the symptoms of coarctation have developed in case of false negative cases, improve the rates of nursery admission, allow earlier initiation of breastfeeding, and decrease medicalization for those neonates with prenatal low-risk CoA suspicion and unconfirmed CoA upon birth (34).

However, our retrospective analysis has also highlighted the importance of proper monitoring of false positive cases of CoA because their postnatal course may still be complicated due to prenatally undetected congenital malformations or transition problems as already described by Van Nisselrooij et al. (23). In our study population, 46% of neonates that had not developed CoA after birth were diagnosed with a cardiac defect or a genetic syndrome that had not been diagnosed *in utero* or with PNPH.

Overall, 20% of all non-CoA cases coped with temporary self-limiting neonatal PNPH that needed respiratory support during the neonatal observation. The prevalence of PNPH in this group of neonates was higher than that reported in unselected cohorts (1.9 per 1000 live births) (22). As previously hypothesized, the altered hemodynamics in fetal life, with right ventricular dominance and increased flow entering the pulmonary circulation, might have an effect on pulmonary vascular development, causing an abnormal transition phase from fetus to newborn and the evidence of PNPH (22, 23).

Therefore, we suggest that parents should be alerted at the time of prenatal counseling of the possible occurrence of other minor or major defects or potential transition problems, whenever there is evidence of significant cardiovascular disproportion, even if CoA suspicion is not confirmed after birth.

There are some limitations to our study. The first is the retrospective design and the small number of included cases. The second limitation is that this study was not planned to evaluate the efficacy of a fetal cardiac screening program but to assess the feasibility of fetal echocardiography for diagnosing CoA, especially during the third trimester of pregnancy. Therefore, we did not report the cases of CoA undiagnosed at prenatal screening and detected only postnatally. We are well aware that it will be necessary to validate our results in future studies. A large multicenter prospective study including all fetuses with CoA suspicion is needed to evaluate the actual diagnostic performance of fetal echocardiography in the diagnosis of CoA.

## Conclusion

The current criteria for diagnosing CoA *in utero* allow accurate diagnosis of most severe CoA cases, but the milder ones are still difficult to be detected and the rate of false positives remains relatively high. A combination of anatomic and functional echocardiographic parameters might help in stratifying the risk of CoA, and the prenatal detection rate of CoA may improve when a multiple criteria prediction model is adopted. We proposed the aortic valve and the transverse aortic arch diameter z-score as predictors of CoA after birth. In addition, neonates without CoA deserve proper monitoring at birth because prenatal evidence of a cardiovascular discrepancy between the right and left cardiac structures has an inherent risk for additional morbidity postnatally.

## Data availability statement

The raw data supporting the conclusions of this article will be made available by the authors, without undue reservation.

## Ethics statement

The studies involving human participants were reviewed and approved by Regione Liguria Ethical Board. Written informed consent to participate in this study was provided by the participants' legal guardian/next of kin.

## Author contributions

GT and MM conceptualised and designed the study, drafted the initial manuscript, and reviewed and revised the manuscript. GT, MM, and LM collected data. MC carried out the statistical analysis and critically reviewed and revised the manuscript. DP, SB, and GD critically reviewed the manuscript for important intellectual content. All authors approved the final manuscript as submitted and agree to be accountable for all aspects of the work.

## Conflict of interest

The authors declare that the research was conducted in the absence of any commercial or financial relationships that could be construed as a potential conflict of interest.

## Publisher's note

All claims expressed in this article are solely those of the authors and do not necessarily represent those of their affiliated organizations, or those of the publisher, the editors and the reviewers. Any product that may be evaluated in this article, or claim that may be made by its manufacturer, is not guaranteed or endorsed by the publisher.
